# Supplementary material for: Alteration of the Dopamine Receptors’ Expression in the Cerebellum of the Lysosomal Acid Phosphatase 2 Mutant (Naked–Ataxia (NAX)) Mouse
Source: Int J Mol Sci. 2020 Apr 21;21(8):2914. doi: 10.3390/ijms21082914 (PMC7215910; doi:10.3390/ijms21082914)
Supplement: Supplementary file 1 [file ijms-21-02914-s001.pdf]

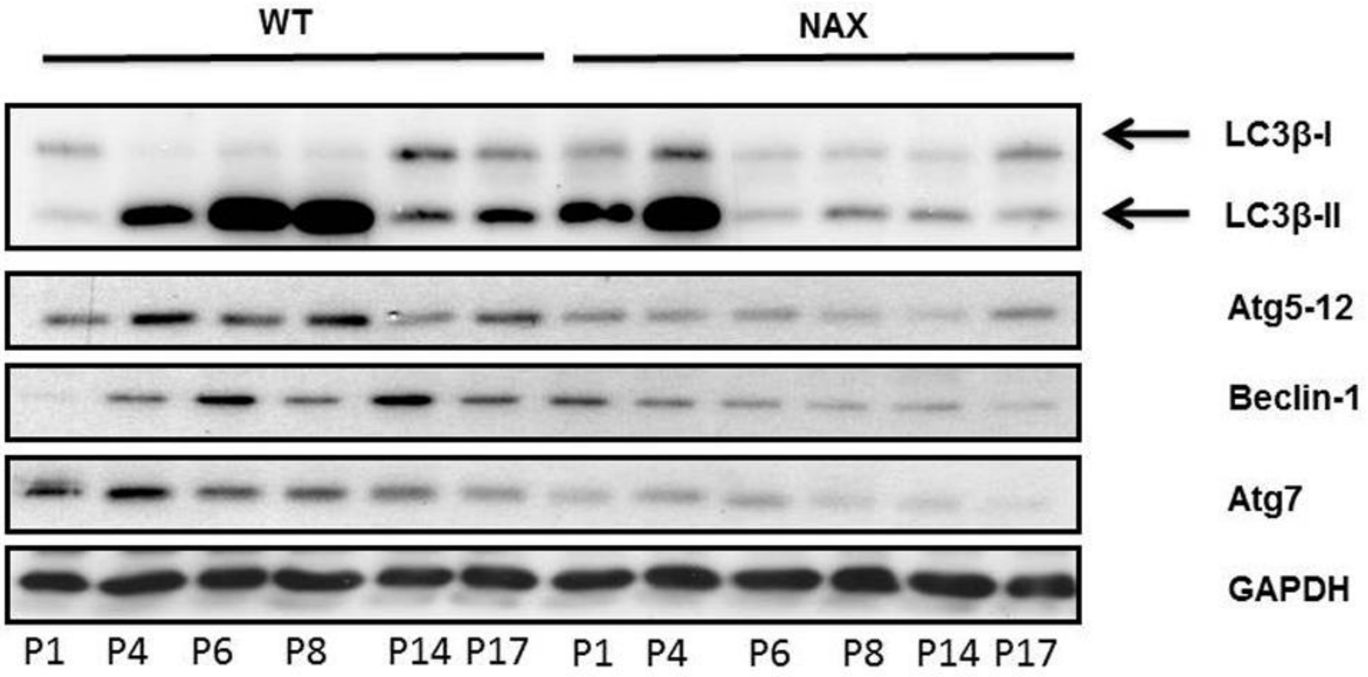

Supplementary Figure. Western blot analysis of autophagy pathway during postnatal cerebellar development. *ACP2* deletion mutation downregulates autophagy machinery in the cerebellum. Total cell lysates from the cerebellum of wild-type and Acp2 point-mutated mice have been prepared for the indicated time-points after the birth (P1, P4, P6, P8, P14, and P17), and immunoblots were arranged for autophagy markers (LC3β, Atg5-12, Beclin1, and Atg7). Our results showed that an *ACP2* deletion mutation decreased LC3β lipidation, Atg5 and Atg12 conjugation, Beclin-1 expression, and Atg7 expression. Protein loading was confirmed using GAPDH. Experiment was repeated using 2 different series of mice.
